# The tongue of the red-eared slider (*Trachemys scripta elegans*): morphological characterization through gross, light, scanning electron, and immunofluorescence microscopic examination

**DOI:** 10.1186/s12917-024-03879-2

**Published:** 2024-02-03

**Authors:** Mohamed A. M. Alsafy, Nermin K. A. El-sharnobey, Samir A. A. El-Gendy, Mohamed A. Abumandour, Basma G. Hanafy, Samar M. Ez Elarab, Ahmed M. Rashwan

**Affiliations:** 1 Anatomy and Embryology Department, Faculty of Veterinary Medicine, Alexandria University, Abees 10th, Alexandria, 21944 Egypt; 2 Department of Histology and Cytology, Faculty of Veterinary Medicine, Alexandria University, Abees 10th, Alexandria, 21944 Egypt; 3 Department of Anatomy and Embryology, Faculty of Veterinary Medicine, Damanhour University, Damanhour, 22511 Egypt; 4 Laboratory of Life science frontiers, Center for iPS Cell Research and Application (CiRA), Kyoto University, 53 Kawahara-cho, Shogoin, Sakyo-ku, Kyoto, 606-8507 Japan

**Keywords:** Turtles, Tongue, Light and scanning electron microscopy, Immunofluorescence microscopy

## Abstract

The red-eared slider (*Trachemys scripta elegans*) is renowned for its remarkable adaptations, yet much of its complex biology remains unknown. In this pioneering study, we utilized a combination of gross anatomy, scanning electron microscopy (SEM), light microscopy, and immunofluorescence techniques to examine the tongue’s omnivorous adaptation in this species. This research bridges a critical knowledge gap, enhancing our understanding of this intriguing reptile. Gross examination revealed a unique arrowhead-shaped tongue with a median lingual fissure and puzzle-piece-shaped tongue papillae. SEM unveiled rectangular filiform, conical, and fungiform papillae, with taste pores predominantly on the dorsal surface and mucous cells on the lateral surface of the papillae. Histologically, the tongue’s apex featured short rectangular filiform and fungiform papillae, while the body exhibited varying filiform shapes and multiple taste buds on fungiform papillae. The tongue’s root contained lymphatic tissue with numerous lymphocytes surrounding the central crypt, alongside lingual skeletal musculature, blood and lymph vessels, and Raffin corpuscles in the submucosa. The lingual striated muscle bundles had different orientations, and the lingual hyaline cartilage displayed a bluish coloration of the ground substance, along with a characteristic isogenous group of chondrocytes. Our research represents the first comprehensive application of immunofluorescence techniques to investigate the cellular intricacies of the red-eared slider’s tongue by employing seven distinct antibodies, revealing a wide array of compelling and significant findings. Vimentin revealed the presence of taste bud cells, while synaptophysin provided insights into taste bud and nerve bundle characteristics. CD34 and PDGFRα illuminated lingual stromal cells, and SOX9 and PDGFRα shed light on chondrocytes within the tongue’s cartilage. CD20 mapped B-cell lymphocyte distribution in the lingual tonsil, while alpha smooth actin (α-SMA) exposed the intricate myofibroblast and smooth muscle network surrounding the lingual blood vessels and salivary glands. In conclusion, our comprehensive study advances our knowledge of the red-eared slider’s tongue anatomy and physiology, addressing a significant research gap. These findings not only contribute to the field of turtle biology but also deepen our appreciation for the species’ remarkable adaptations in their specific ecological niches.

## Introduction

Turtles are among the top animals recently used as exotic pets for their unique body shapes and sizes, long life span, unique appearance, calmness, and ease of care and handling [1, 2]. Over time, turtles have played an essential role in ecosystems worldwide since around 220 million years ago, as they inhibited freshwater and marine ecosystems [3].

Red-eared sliders are a type of turtle whose scientific name is *Trachemys scripta elegans*, pond sliders, or red-eared terrapins [4] and is a native indigenous species in the United States [5]. However, with time, they began to crawl to other ranges outside their natural range. They were introduced to some places and other countries within Europe, Asia, Africa, and Australia via online marketing, as the increasing demand for turtles as pets led to the emergence and presence of new species that weren’t widespread [6, 7]. This gives a good reason for the spread of red-eared sliders in Egyptian pet shops or local markets, especially in Alexandria. It has been a noticeable phenomenon to sell turtles as pet animals.

The red-eared slider is an ideal opportunistic omnivorous reptile [8] that eats a wide range of invertebrates like shrimps, shellfish, crabs, snails, and insects; vertebrates like fish, rodents, frogs, lizards, birds, snakes, and some aquatic plants such as ferns, algae, and seed plants [9]. The red-eared slider is a freshwater terrapin that can withstand various habitats, such as rivers, ditches, swamps, streams, and ponds [9, 10]. Although they prefer these quiet water sources, they can tolerate and adapt to even brackish water with a salinity of 5.3 to 14.6 [11]. The red-eared slider might reach a carapace length of more than 16 inches (40 cm), but the average size is between 6 and 8 inches (equivalent to 15 to 20 cm), accompanied by an average mass of 3.2 kg [12]. Generally, turtles are a focus point for many researchers due to their unique body characteristics and the multiplicity and variations of their shapes and sizes worldwide [13, 14]. The most attractive points for researchers were structures related to longevity and reproduction [15, 16], outer shell and carapace morphology [17, 18], neck movement and retraction [19], and feeding habits and oropharyngeal adaptation in different species [20, 21].

The lingual epithelium of turtles displays considerable variation in the structure and distribution of lingual papillae across different turtle species. Substantial distinctions in the morphological and structural characteristics of turtle tongues can be attributed to factors such as dietary preferences, food processing, physical adaptations, and diverse environmental conditions [21]. Despite these variations, the tongues of turtles are primarily composed of non-keratinized cells filled with secretory granules. The dorsal lingual epithelium is characterized by a stratified squamous epithelium abundant in mucous cells and features a multitude of mechanically diverse papillae, notably filiform papillae. Additionally, the presence of abundant gustatory papillae, such as fungiform papillae housing taste buds, has been documented [22–24]. Our investigation aims to delve into the distinct protein localizations and functions within these papillae of the red-eared slider’s tongue through the utilization of immunofluorescence techniques.

Vimentin is a protein that forms elongated, slender fibers known as intermediate filaments within cells. It is found in a wide range of cell types, including not only stromal cells, which provide support and surround tissues and organs, but also in epithelial cells, fibroblasts, endothelial cells, and various cells derived from the mesenchyme [25]. Furthermore, Vimentin is also used as a marker to visualize taste buds [26]. Synaptophysin, a well-studied neuronal protein integral to synaptic vesicles, has undergone recent investigations that have unveiled its presence and potential roles within taste bud cells, marking a significant development in the understanding of taste perception [27, 28]. CD34, a widely employed transmembrane glycoprotein, is a cell surface marker for different cell types, including fibroblasts, stem cells, and progenitor cells [29]. In identifying telocytes within the stroma of the human tongue, CD34/PDGFRα double immunofluorescence has emerged as a valuable immunohistochemical marker [30]. SOX9 expression is a hallmark of chondroprogenitor cells and signifies their commitment to the cartilage lineage [31]. SOX9’s influence extends to all stages of chondrocyte development, from initial specification to terminal differentiation [32]. PDGFRα also plays a pivotal role in forming chondrocyte progenitors in craniofacial cartilage development, which is essential for cartilage development [33]. Therefore, we decided to perform double immunofluorescence staining of SOX9 and PDGFRα to visualize chondrocytes within the lingual cartilage of the red-eared slider. CD20, also known as B-lymphocyte antigen CD20, is a cell surface glycoprotein encoded by the MS4A1 gene and identified as a B-cell-specific marker [34]. α-SMA, a well-known protein that plays a crucial role in cellular biology, has garnered significant attention due to its specialized expression in smooth muscle cells and myofibroblasts [35].

The current study focused on the tongues of recently introduced pet turtles in Egypt, specifically the red-eared slider. We conducted a comprehensive anatomical and histological examination using various techniques, including gross, light, scanning electron microscopy, and immunofluorescence analysis. Our groundbreaking research involved using seven distinct antibodies to explore the red-eared slider turtle’s tongues thoroughly. These antibodies were invaluable tools for uncovering the intricate mechanisms governing tongue function and its remarkable adaptations to different ecological conditions. Notably, no previous studies have employed immunofluorescence techniques in any turtle species. This pioneering research aimed to fill existing knowledge gaps and shed new light on the fascinating world of turtle tongues.

## Materials and methods

### Animals

The current study was conducted on ten adult red-eared sliders with carapace lengths of 23.4 to 25.1 cm and a weight ranging from 1.41 to 1.73 kg. They were collected from the local pet shops in Alexandria, Egypt. All turtles were transported to our anatomical labs in travel pet cages within 2 h and kept for a while to ensure they were healthy and free from oral abnormalities or injuries. The red-eared sliders were kept in an aquarium with small and large stones and offered a variety of commercial pellets, small fish such as sardines, and some vegetables. For further examinations of the tongue, each red-eared slider firstly was anesthetized with (2 mg/kg) xylazine intramuscular [36] and then was euthanized with a lethal dose of ketamine (100 mg/kg) intramuscular, unconsciousness, painless, then brain death occurred 90 s after the ketamine injection, the death confirmed via the absence of reflexes, movement, heartbeat, and absence of cardiac electrical activity, this method of euthanasia produced no histopathologic changes in the tissue specimens studied, even though death came quickly [37–40]. After profound narcosis, all animals were decapitated.

### Gross morphological examination

All heads were divided entirely horizontally from the mouth to the esophagus in two parts. Then, the tongue was photographed using (a Canon EOS 2001) digital camera.

### Light microscopy examination

Five fresh tongue samples, each measuring 0.5 × 1 cm, were collected from the tongue’s apex, body, and root. The collected samples were immersed in 10% phosphate-buffered formaldehyde and left to fix overnight. Subsequently, the samples were washed with phosphate buffer saline for one day at 4 °C. After fixation, the tongue tissues underwent a dehydration process with ascending grades of ethanol, starting with 50%, followed by 70%, 80%, and 90%, each for 15 min, and a final wash with 100% ethanol three times, each for 10 min. Following dehydration, the samples were cleared with xylene and subsequently embedded in soft, and hard paraffin wax (respectively). Transverse sections, each measuring 4 μm, were obtained using a microtome and then mounted on glass slides. The tissue sections were stained with Mayer’s hematoxylin and eosin stain (H&E) for general comprehensive histological examinations following established protocols outlined by [41]. Masson’s trichrome was also used for visualizing collagen fiber and muscle fiber [42] and the Periodic Acid Schiff (PAS) technique for identifying mucopolysaccharides and neutral mucin [43]. The slides were examined by an Optica Italian microscope. The photomicrographs were taken by an Optica camera [44].

### SEM examination

Five fresh tongues were fixed in a buffer solution comprising 2% formaldehyde, 1.25% glutaraldehyde, and 0.1 M sodium cacodylate at pH 7.2 and 4℃. Following fixation, the samples underwent washing in 0.1 M sodium cacodylate containing 5% sucrose, tannic acid processing, and subsequent dehydration in increasing grades of ethanol (15 min each in 50%, 70%, 80%, 90%, 95%, and 100% ethanol). The specimens were then dried in carbon dioxide, affixed to stubs with colloidal carbon, and coated with gold–palladium in a sputtering device. Examination and photography of the specimens were conducted using a JEOL JSM-IT200 scanning electron microscope at 15 kV at the Electron Microscope Unit, Faculty of Science, Alexandria University. This methodology closely aligns with protocols employed in previous studies [45].

### Immunofluorescence microscopy examination

We employed seven distinct antibodies to comprehensively investigate the cellular landscape of the red-eared slider’s tongue. Briefly, 5-µm paraffin sections were deparaffinized with Histo-Clear, then rehydrated through decreasing ethanol concentrations and rinsing with distilled water. Antigen retrieval was done using autoclaved Target Retrieval Solution pH 6.0 (S169984, Dako, USA), followed by PBS washing. Permeabilization was achieved by immersing slides in 0.2% Triton-PBS for 20 min. To minimize nonspecific reactions, sections were blocked with protein blocks (Dako, Vector Laboratories, USA) for 30 min at room temperature. Subsequently, sections were incubated overnight at 4 °C with primary antibodies (Table 1). Following PBS washing, sections were incubated for 60 min with secondary antibodies (Table 2). Finally, slides were counterstained with DAPI using Vectashield Antifade Mounting Medium (Vec-H-1200-10, Vector Laboratories, USA), and images were captured using a BZ-9000E HS All-in-One Fluorescence Microscope (Keyence) [46].

**Table 1:** Primary antibodies employed in this current investigation

Antibodies	Species	Dilution	Source, catalogue no #
1. Vimentin	Goat	1:1000	Dallas, Texas, USA, Santa Cruz Biotechnology, Cat# sc-7558
2. Synaptophysin	Rabbit	1:100	Waltham, Massachusetts, USA, Thermo Fisher Scientific, Cat# # 0407-2
3. CD34	Rat	1:100	San Diego, California, USA, eBioscience, Cat# 14-0341-81.
4. PDGFRα	Rabbit	1:100	Danvers, Massachusetts, USA, Cell Signaling Technology, Cat# 3174
5. SOX9	Goat	1:50	Dallas, Texas, USA, Santa Cruz Biotechnology, Cat# sc-17,340
6. CD20	Mouse	1:250	Waltham, Massachusetts, USA, Thermo fisher scientific, Cat# MA5-13141
7. α-SMA	mouse	1:50	Cambridge, UK, Abcam, Cat# ab7817

**Table 2:** Secondary antibodies employed in this current investigation

Conjugate	Species	Antigen	Dilution	Supplier
1. Alexa fluor 488	Donkey	Rabbit IgG	1:500	Thermo Fisher Scientific Waltham, MA, USA
2. Alexa fluor 488	Donkey	Rat IgG	1:500	Thermo Fisher Scientific Waltham, MA, USA
3. Alexa fluor 488	Donkey	Goat IgG	1:500	Thermo Fisher Scientific Waltham, MA, USA
4. Cyanine 3	Donkey	Rabbit IgG	1:400	Merck Millipore Massachusetts, USA
5. Cyanine 3	Donkey	Mouse IgG	1:400	Merck Millipore Massachusetts, USA

## Results

### Gross anatomy

**Fig. 1:**
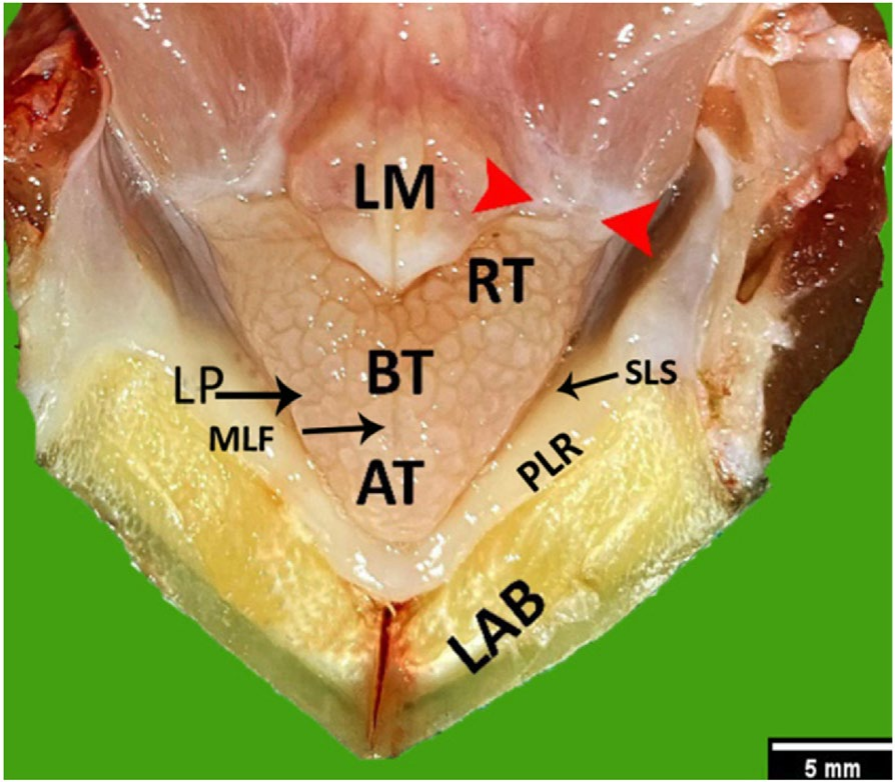
A gross dorsal view of the floor of the oropharyngeal cavity of an adult red-eared slider explains the tongue. Lower alveolar band (LAB), apex of the tongue (AT), the body of the tongue (BT), root of the tongue (RT), lingual papillae (LP), paralingual ridge (PLR), sublingual space (SLS), median lingual fissure (MLF), laryngeal mound (LM) and ligament of the root of the tongue (red arrowheads). Scale bar = 5 mm

The tongue extended from the lower alveolar band to the laryngeal mound and was divided into the apex, body, and root. The apex was pointed anteriorly and firmly attached to the sublingual floor, while the middle of the body featured a median lingual fissure. The root of the tongue appeared as two wings and was fixed caudally by the ligament of the tongue’s root. The tongue papillae had a distinctive puzzle-piece appearance (Fig. 1). The length of the tongue measured 13.04 ± 0.97 mm, and its width varies in three regions, measuring 2.17 ± 0.014 mm at the apex, 6.31 ± 0.028 mm at the body, and 9.57 ± 0.046368 mm at the root of the tongue.

### SEM results

**Fig. 2:**
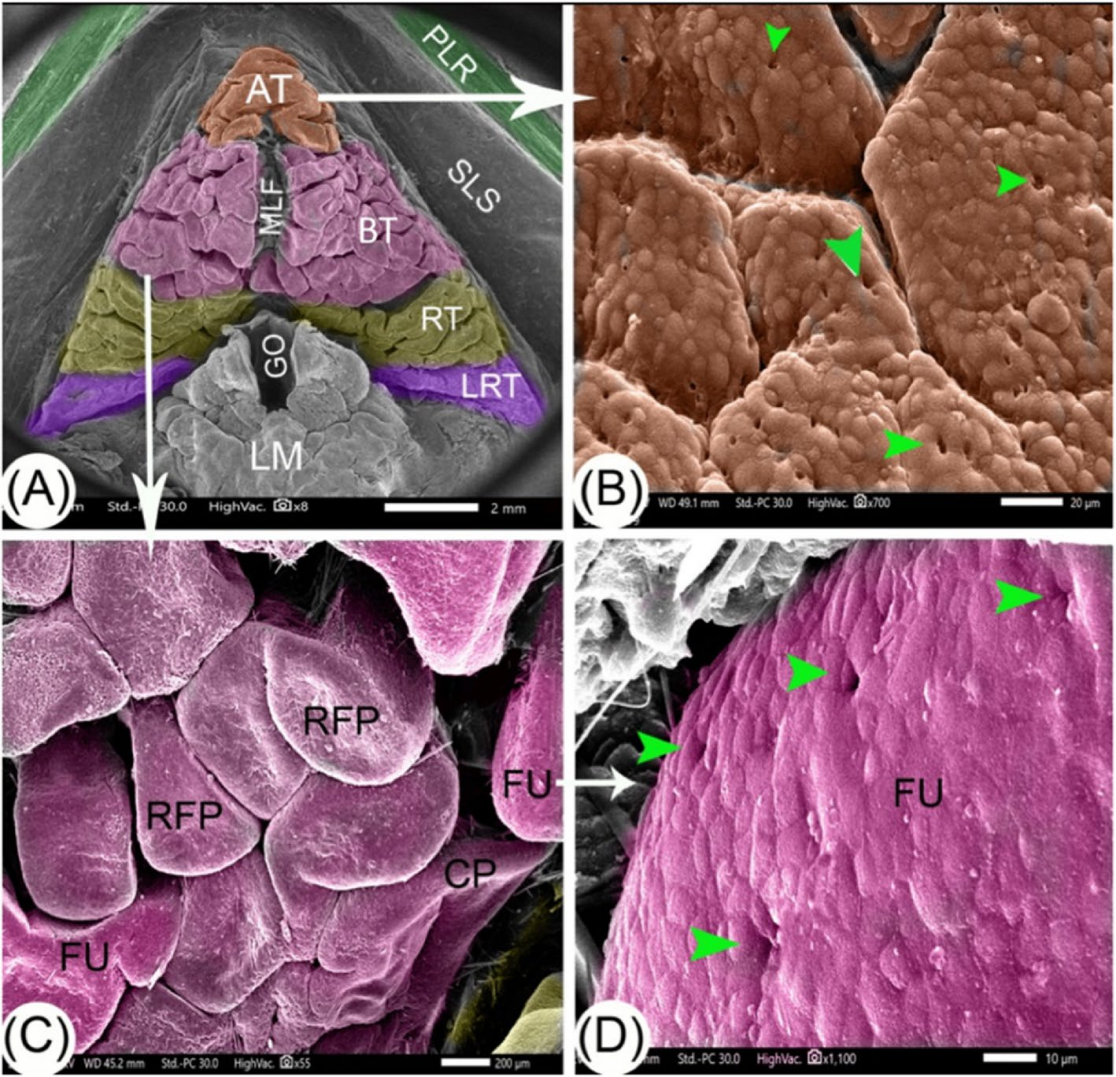
SEM images (Views **A**-**D**) of the tongue of the red-eared slider shows the following: paralingual ridge (PLR), apex of the tongue (AT), the body of tongue (BT), root of tongue (RT), ligament of root of the tongue (LRT), glottis (GO), laryngeal mound (LM), sublingual space (SLS), median lingual fissure (MLF), taste pores (green arrowheads), rectangular filiform papillae (RFP), conical papillae (CP) and fungiform papillae (FU). Scale bars: A = 2 mm, B = 20 μm, C = 200 μm, and D = 10 μm

The tongue exhibited an arrowhead shape with a median lingual fissure (Fig. 2A). At the apex of the tongue, there were ridge-like papillae that were fused closely together and had surface taste pores (Fig. 2B). In the tongue’s body, we observed several ridges of rectangular and conical filiform papillae (Fig. 2C). Particularly noteworthy were the fungiform papillae with taste pores (Fig. 2D). The lingual papillae were directed caudally towards the epiglottis and had the maximum height. The root of the tongue, situated in the caudal part, resembled two wings that pointed caudally. The papillae on the tongue’s root were mainly filiform.

### Histological structure of the tongue of the red-eared slider

**Fig. 3:**
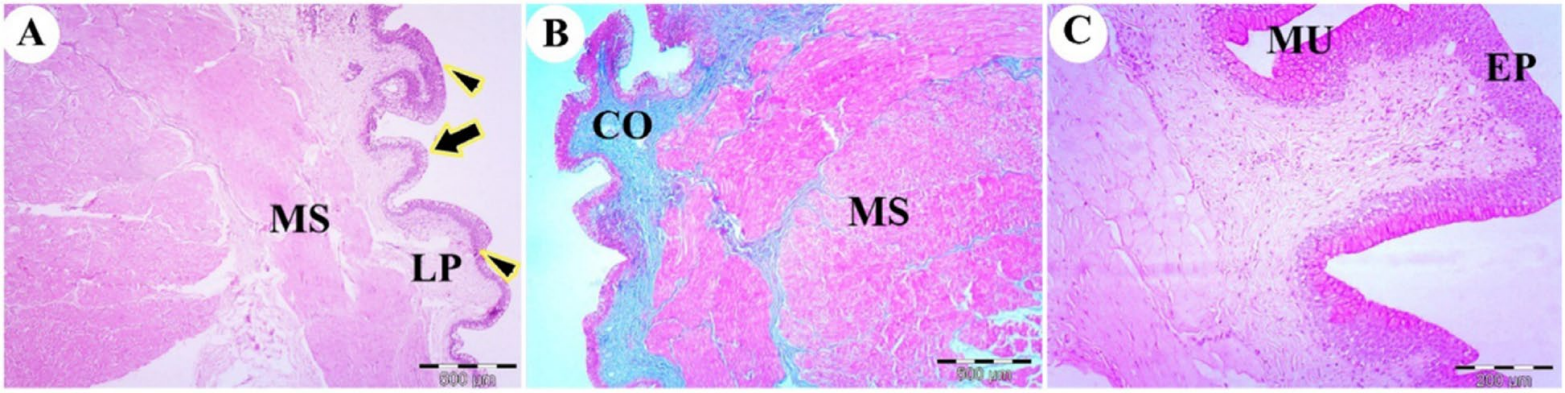
Photomicrographs of the dorsal surface of the tip of the tongue of the red-eared slider. View (**A**) shows the rectangular filiform papillae (arrowheads), fungiform papillae (arrow), lamina propria (LP), and skeletal muscle bundles with different oriantation (MS). (H&E, Mag.40X, bar = 500 μm). View (**B**) shows the collagen fiber of lamina propria (CO) and skeletal muscle (MS). (Masson trichrome, Mag.40X, bar = 500 μm). View (**C**) shows the stratified lingual epithelium (EP) and mucous cells on the lateral surface of the lingual epithelium (MU) (PAS, Mag.40X, bar = 500 μm)

The dorsal mucosal surface of the tip of a red-eared slider’s tongue was formed by stratified squamous epithelium. The outer surface of the tongue’s apex had short, rectangular filiform papillae and dome-shaped fungiform papillae (Fig. 3A). The lamina propria, the core of the lingual papillae, was composed of dense, irregular connective tissue. Well-developed skeletal musculature extended to the tongue’s apex, with collagen fibers detected by Masson’s trichrome stain in the lamina propria (Fig. 3B). There were high numbers of mucous cells on the lateral surface of the lingual filiform papillae that stained positively with periodic acid-Schiff (PAS) (Fig. 3C).

**Fig. 4:**
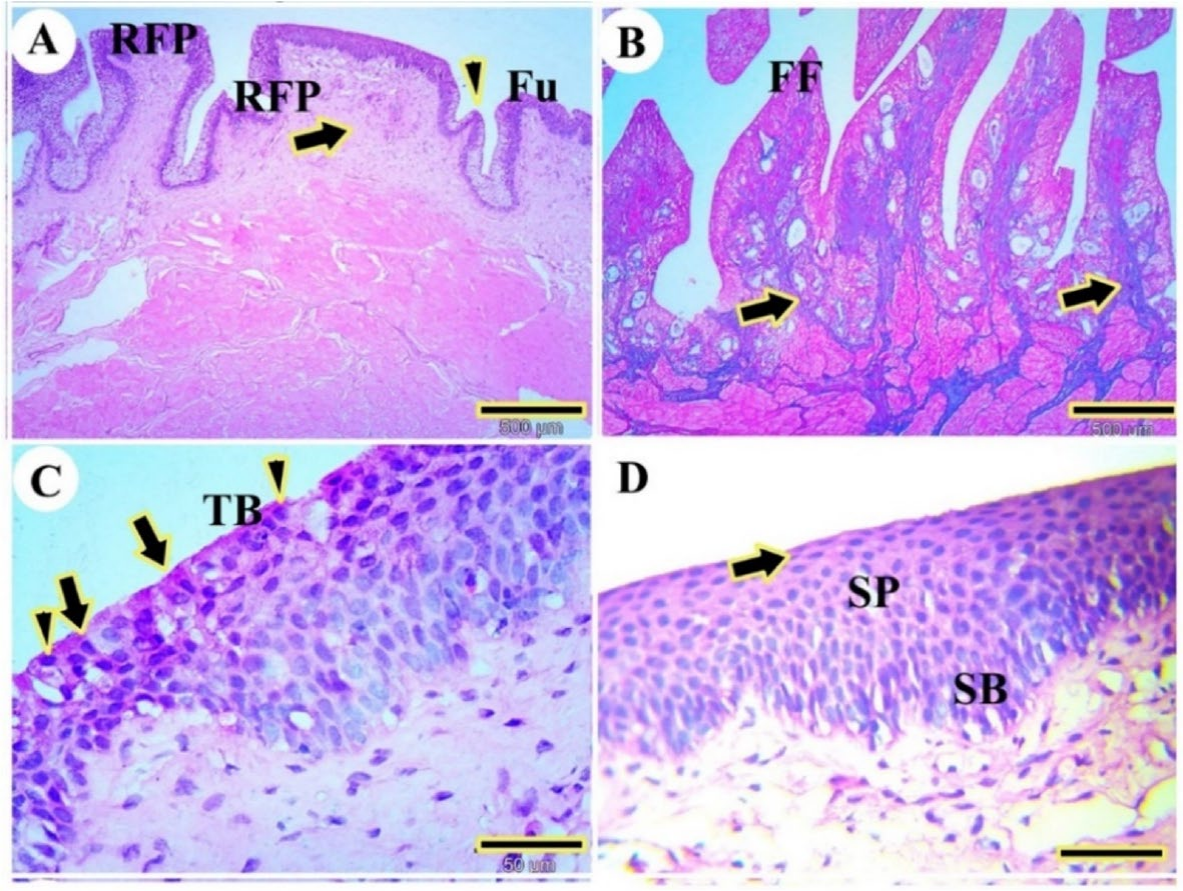
Photomicrographs of the dorsal surface of the body of the tongue of the red-eared slider. View (**A**) shows rectangular filiform papillae (RFP), conical papilla (arrowheads), fungiform papilla (Fu), and lamina propria (arrow). (H&E, Mag.40X, bar = 500 μm. View (**B**) pointed filiform papillae (FF) and collagen fiber (CO). (Masson trichrome, Mag.40X, bar = 500 μm). View (**C**) shows serial taste buds (TB), sensory cells (arrow), and supporting cells (arrowheads) (H&E, Mag.400X, bar = 50 μm). View (**D**) shows stratum basalis (SB), stratum spinosum (SP), and outermost squamous cells layer (arrow). (H&E, Mag.400X, bar = 50 μm)

The tongue body of the red-eared slider turtle featured distinct filiform papillae, classified as mechanical papillae, exhibiting various shapes, including rectangular and conical forms (Fig. 4A), as well as pointed papillae (Fig. 4B). Additionally, numerous gustatory papillae, identified as fungiform papillae, were present (Fig. 4A). These papillae possessed a lamina propria composed of a core of dense, irregular connective tissue (Fig. 4A) and collagen fibers (Fig. 4B). Within the fungiform papillae, multiple taste buds were present on the apical surface, consisting of bipolar sensory nerve cells and supporting cells (Fig. 4C). The dorsal mucosal surface was characterized by non-keratinized stratified squamous epithelium organized into the outer squamous cell layer, stratum spinosum, and stratum basalis layers, situated on a corrugated basement membrane. The outer squamous cell layer contained acidophilic squamous cells; the stratum spinosum comprised acidophilic polyhedral cells; and the stratum basalis was composed of basophilic columnar cells (Fig. 4D).

**Fig. 5:**
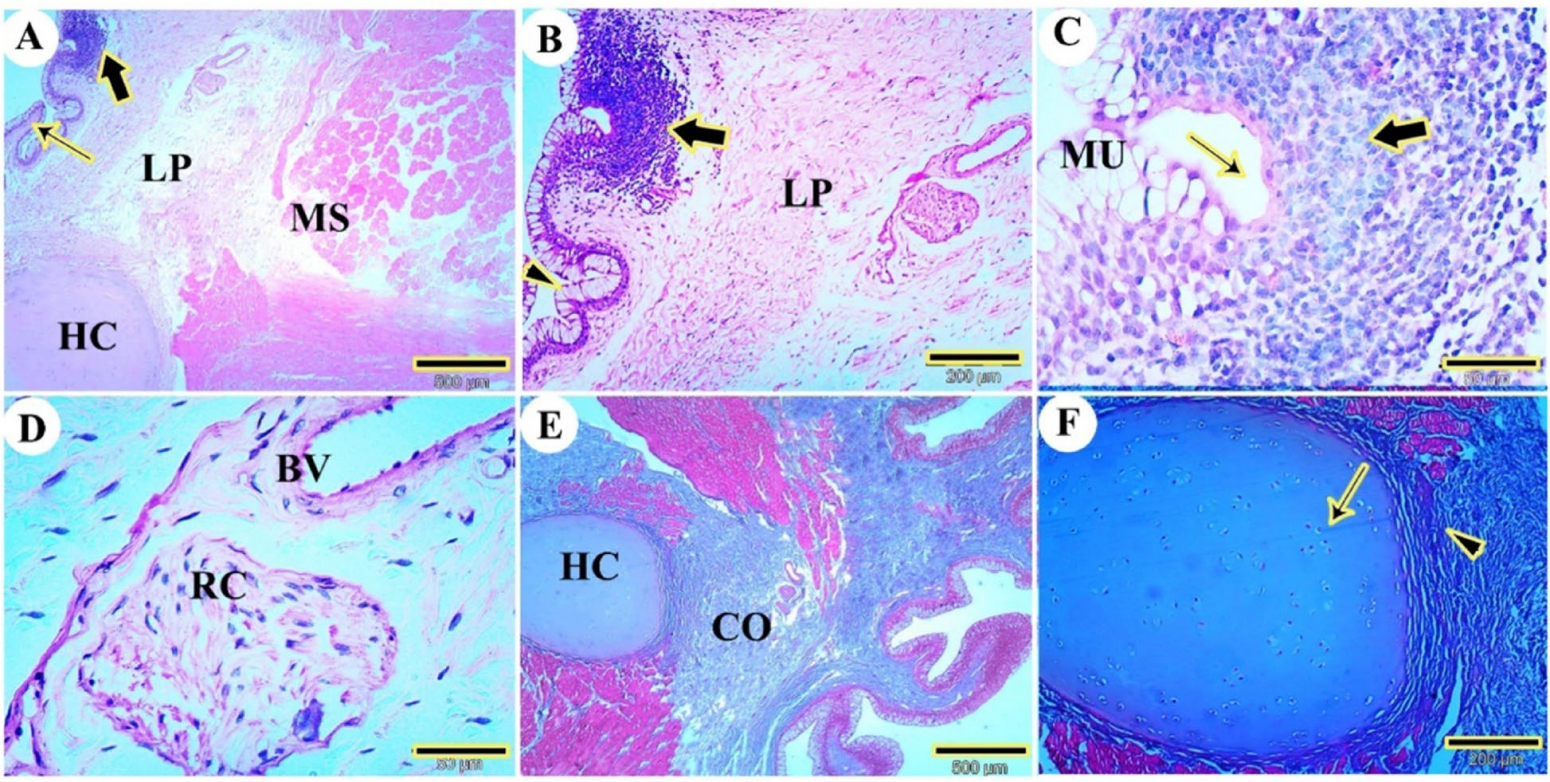
Photomicrographs of the dorsal surface of the root of the tongue of the red-eared slider. Views (**A** and **B**) show the pointed filiform papillae (thin arrow), lymphoid-associated tissue (thick arrow), lamina propria (LP), lingual musculature (MS), lingual hyaline cartilage (HC), and conical papillae (arrowheads) (H&E, Mag.40, bar = 500, and 200 μm respectively). View (**C**) shows the mucosal associated lymphoid tissue of the tongue (thick arrow), crypt (thin arrow), and mucous surface cell (MU) (H&E, Mag.400X, bar = 50 μm). View (**D**) shows Ruffini corpuscle (RC) and blood vessels (BV) (H&E, Mag.400X, bar = 50 μm). Views (**E** and **F**) show the collagen fiber core of lamina propria (CO), skeletal muscles (thick arrow), hyaline cartilage (HC), perichondrium (arrowhead), and chondrocytes (thin arrow). (Masson trichrome, Mag.40X, bar = 500 and 200 μm respectively)

The root of the tongue retained filiform and conical papillae. The lymphatic tissue in the lingual tonsil was organized as lymphoid tissue in the submucosa of the tongue’s root, extending to the mucosal surface and featuring a distinct crypt (Fig. 5A, B). The epithelium adjacent to the lymphoid tissue transformed into lymph-epithelium, infiltrated by a high number of lymphocytes, losing its typical epithelial stratification. The lingual epithelium housed the central crypt (Fig. 5C). The propria submucosa was situated between the lingual mucosal epithelium and the lingual skeletal musculature, with blood vessels, lymph vessels, and Ruffini corpuscles (Fig. 5D). The lingual musculature comprised striated muscle bundles oriented in various directions (Fig. 5E). Within the core of the tongue, there was hyaline cartilage, surrounded by skeletal muscle fibers and collagen fibers. The lingual hyaline cartilage exhibited a bluish coloration of the ground substance and featured a characteristic isogenous group of chondrocytes (Fig. 5F).

### Immunofluorescence results

Our study marks a significant milestone as the first investigation to employ immunofluorescence techniques to examine seven distinct antibodies’ reactions within the tongue of red-eared slider turtles. We utilized a range of antibodies to explore various aspects of the turtle’s lingual tissue, shedding light on its intricate microanatomy and function.

### The distribution of Vimentin in the tongue of red-eared slider turtles

**Fig. 6:**
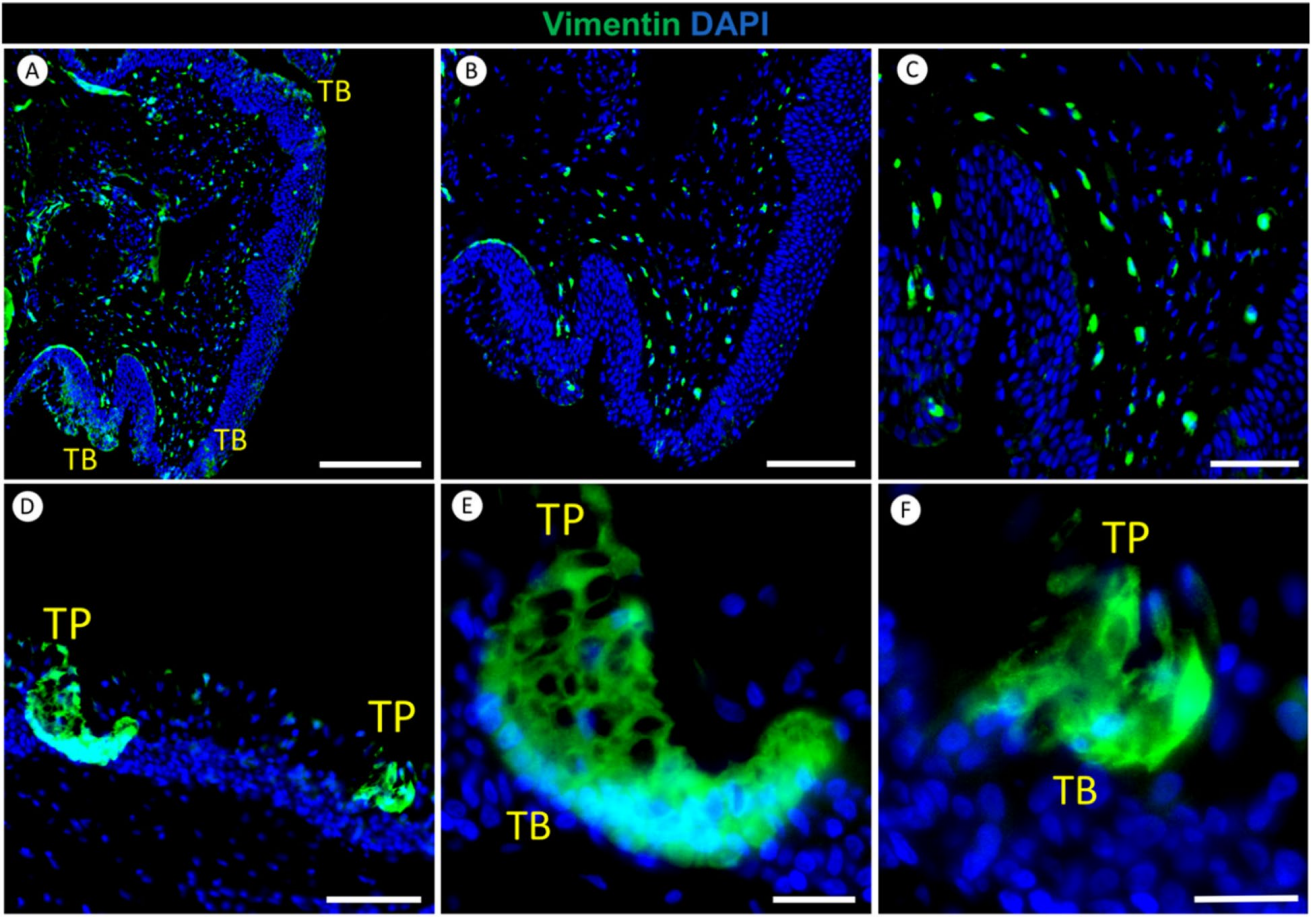
Immunofluorescence staining of Vimentin in the tongue of the red-eared slider. Green indicates Vimentin, while blue represents nuclei counterstained with 4′,6-diamidino-2-phenylindole (DAPI). Panels **A**-**C** illustrate Vimentin expression in the lamina propria, stroma, and taste buds (TB) of the fungiform papilla in the red-eared slider’s tongue. Panel **D**-**F** showcases taste buds (TB) and taste pores (TP) on the epithelial surface of the fungiform papilla in the tongue of the red-eared slider. Scale bars: A = 200 μm, B = 150 μm, C and D = 50 μm, E and F = 25 μm

Vimentin was notably present in three specific locations within the red-eared slider turtle’s tongue (Fig. 6). Firstly, Vimentin expression was observed in the lamina propria beneath the fungiform papillae (Fig. 6A), highlighting its role in supporting the structural framework of the papillae. Secondly, Vimentin expression was detected in the stroma of the fungiform papillae (Fig. 6A, B). Although Vimentin expression in the stroma was relatively limited, it emphasizes the significance of vimentin-expressing fibroblasts and supporting cells in providing structural support to the tongue tissue. Lastly, Vimentin was found to be expressed in taste bud cells, including observable taste pores (Fig. 6A, D, E, F).

### Sensory neural network and taste perception mechanisms in the lingual tissue of the red-eared slider turtles

**Fig. 7:**
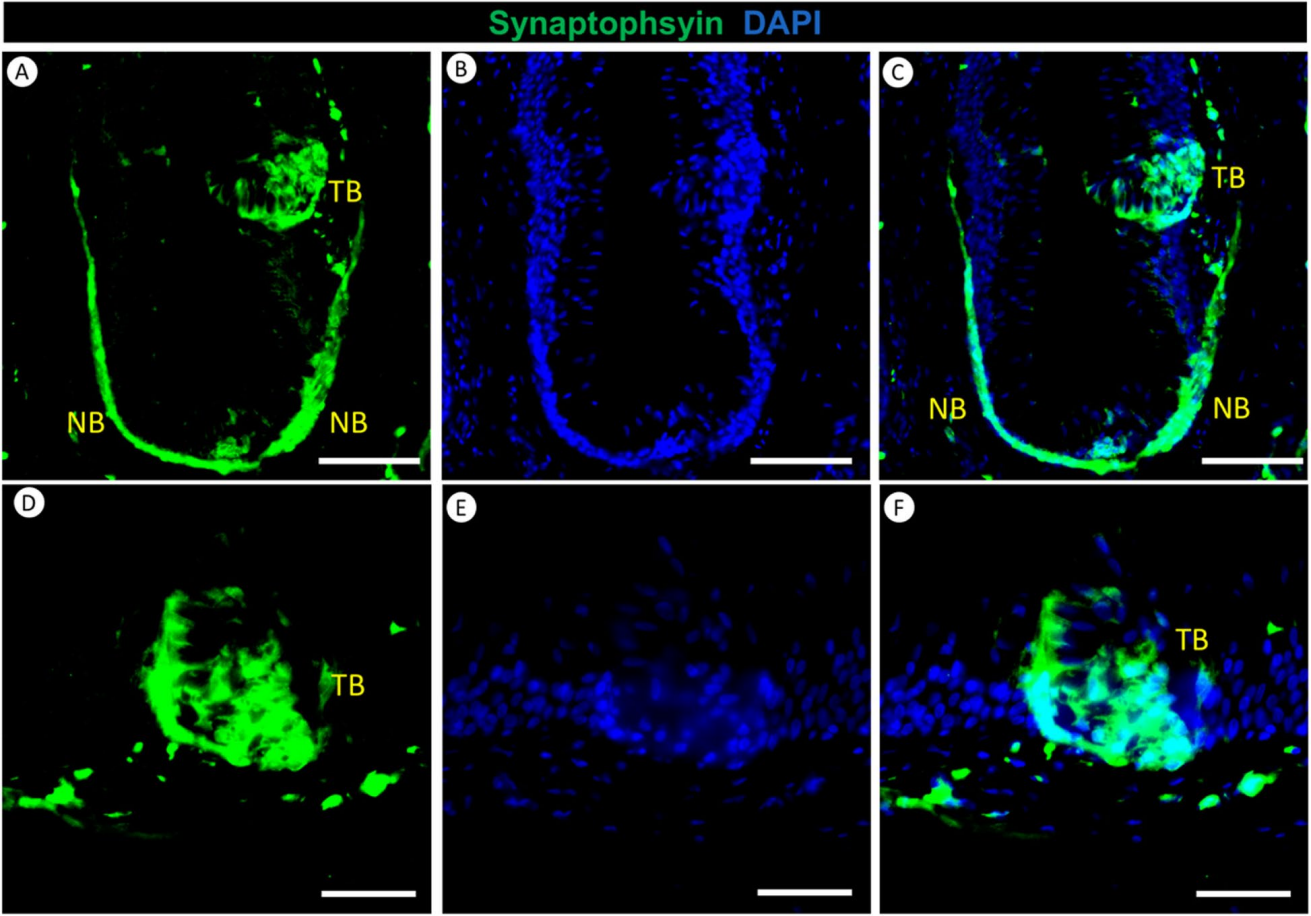
Immunofluorescence staining of Synaptophysin (Green) and DAPI (Blue) in the red-eared slider’s tongue. Panel **A**-**F** illustrates the presence of Synaptophysin within nerve bundles (NB) located in the lamina propria of fungiform papillae, as well as its expression within the taste bud cells (TB) situated on the epithelial surface of the fungiform papilla in the tongue of the red-eared slider. Panels **D**-**F** provide a closer view, showing a higher magnification of Synaptophysin expression within the taste bud cells on the epithelial surface of the fungiform papilla in the red-eared slider’s tongue. Scale bars: A-C = 100 μm, D-F = 50 μm

We used synaptophysin staining as a powerful tool to visualize neural structures in the lingual tissue of the red-eared slider turtle. Synaptophysin was identified in two distinct locations, revealing the existence of a highly organized sensory neural network within the red-eared slider’s lingual tissue (Fig. 7). Firstly, synaptophysin was prominently present in the nerve bundles encircling the papillae (Fig. 7A). This finding underscores the critical role of synaptophysin in mediating neural signaling within the tongue. Secondly, synaptophysin was also found within taste buds (Fig. 7A), implying its participation in synaptic communication within these sensory domains.

### Presence and significance of CD34 and PDGFRα positive cells in papillae of the red-eared slider turtle tongue

**Fig. 8:**
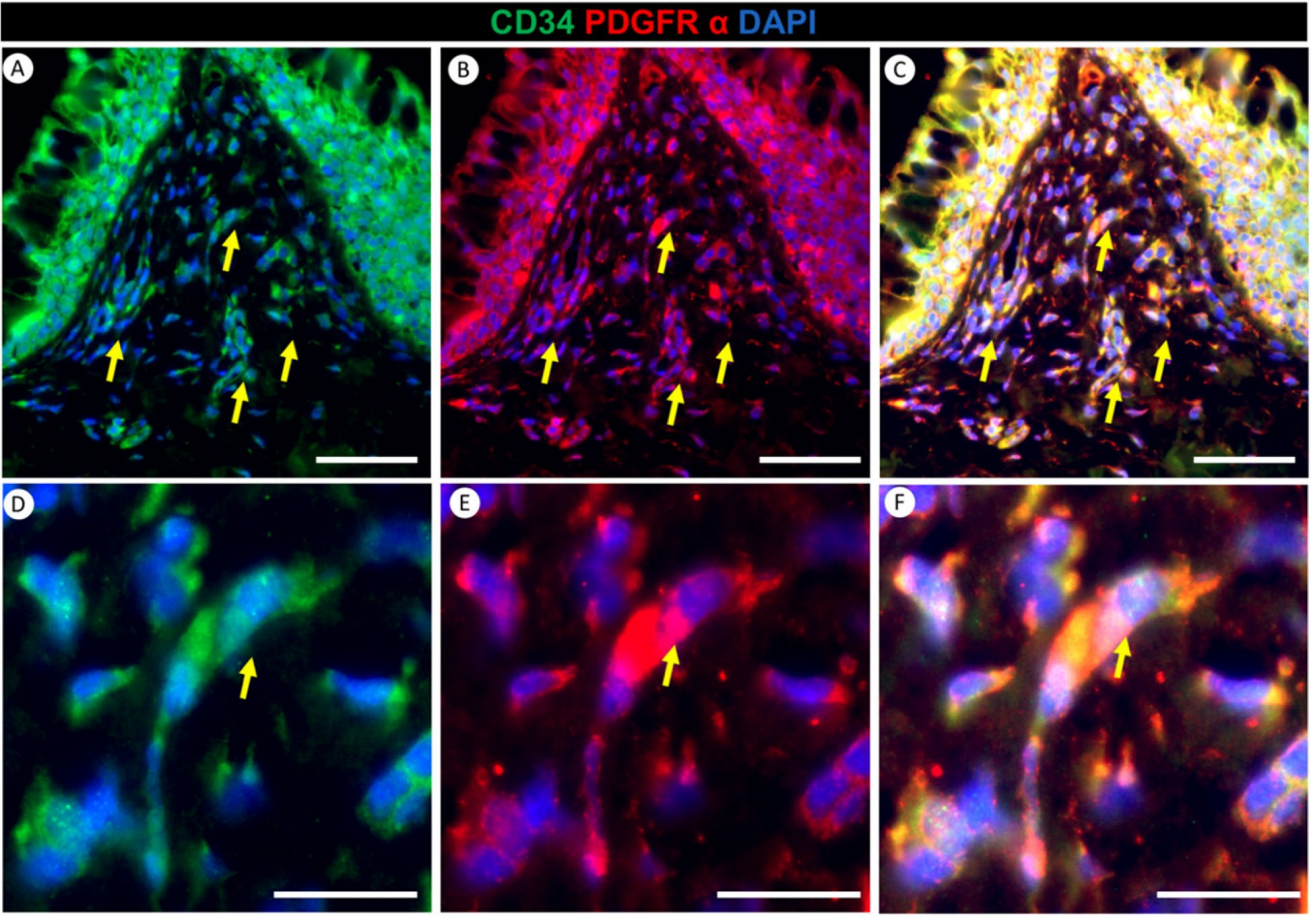
Double immunofluorescence staining for CD34 (Green) and PDGFRα (Red) with DAPI (Blue) in the filiform papilla of the red-eared slider. Panels **A** and **B** demonstrate positive CD34 and PDGFRα expression, respectively, within the lamina propria and stroma of the filiform papilla (yellow arrows). Panel **C** shows the co-expression of CD34 and PDGFRα. Panels **D**-**F** provide a higher magnification view of CD34 and PDGFRα-positive cells (yellow arrow) within the lamina propria of the filiform papilla in the red-eared slider. Scale bars: A-C = 50 μm, D-F = 20 μm

We identified the presence of CD34 and PDGFRα-positive cells within the lamina propria and stroma of papillae during our investigation of lingual tissue from the red-eared slider turtle (Fig. 8A). This finding suggests their active involvement in the structural and regulatory aspects of these papillae. Notably, we observed co-expression of CD34 and PDGFRα, as indicated by the yellow coloration (Fig. 8C, F), signifying potential interactions and crosstalk between these cell populations. This highlights their collaboration in maintaining the lingual microenvironment. Upon closer examination at higher magnification, the CD34 and PDGFRα-positive cells in the Red-eared slider turtle’s tongue revealed an elongated spindle-shaped morphology with central nuclei, resembling the characteristic shape of telocytes. These telocytes were particularly prominent within the lamina propria of the papillae (Fig. 8D).

### Characterization of SOX9 and PDGFRα expression in hyaline cartilage of the red-eared slider turtle tongue

We detected the presence of longitudinal hyaline cartilage in the tongue body and circular hyaline cartilage in the tongue root of red-eared slider turtles. Intrigued by this finding, we endeavored to investigate the chondrocytes residing within the lingual hyaline cartilage. We employed double immunofluorescence staining using SOX9 and PDGFRα markers to examine the chondrocyte cells. Our study represents a pioneering exploration into the molecular composition of hyaline cartilage in the tongue of red-eared slider turtles. Remarkably, there has been a significant dearth of research examining the presence and significance of key markers such as SOX9 and PDGFRα in chondrocytes within this specific cartilage type.

**Fig. 9:**
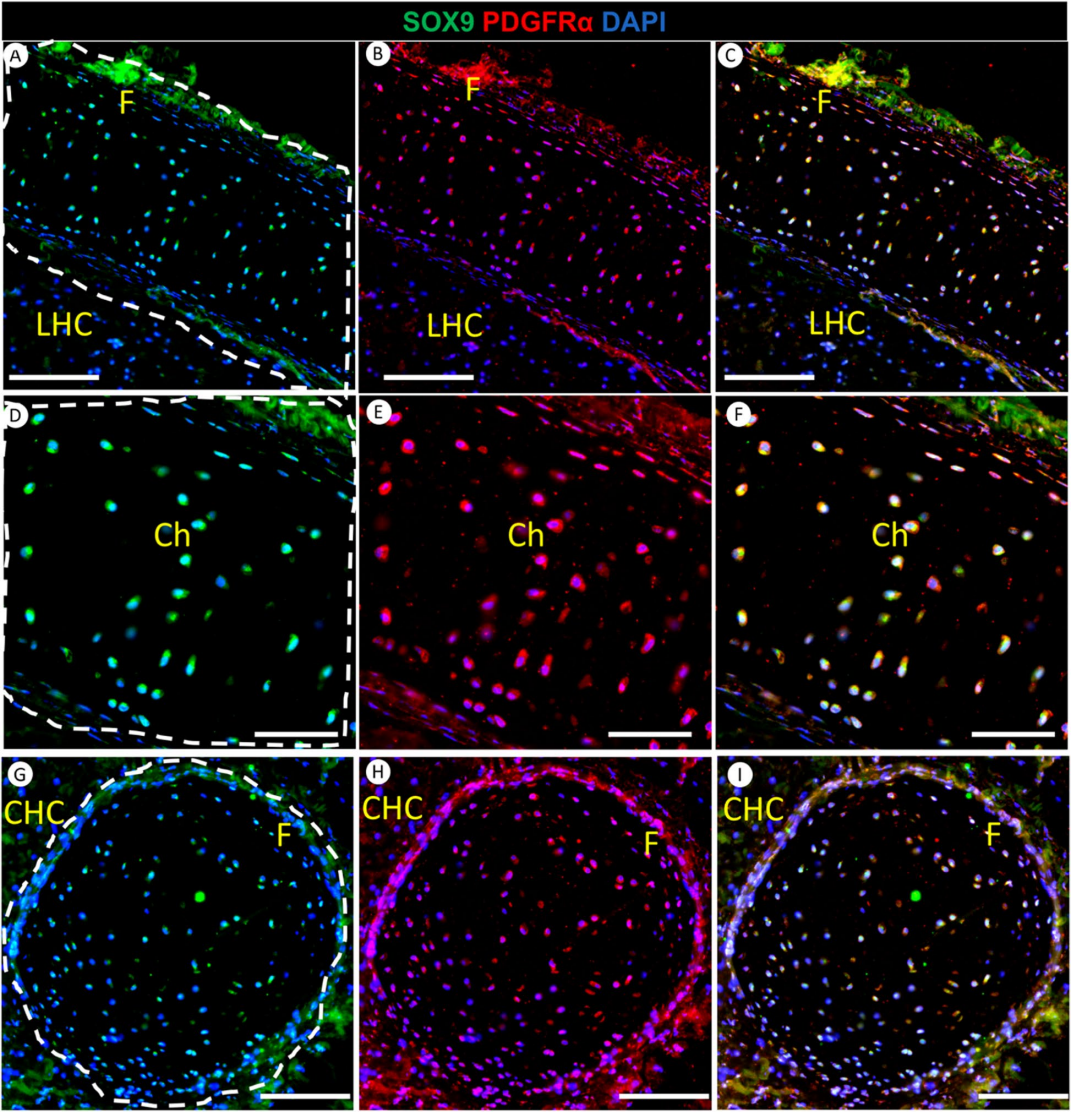
Double immunofluorescence staining for SOX9 (Green) and PDGFRα (Red) with DAPI (Blue) in the tongue root of the red-eared slider. The tongue of the red-eared slider contains longitudinal hyaline cartilage in the body and circular hyaline cartilage in the root. Panels **A**-**C** depict the longitudinal lingual hyaline cartilage (LHC), chondrocytes (Ch), and perichondrium (F). These chondrocytes express SOX9 and PDGFRα. Panels **D**-**F** provide a higher magnification of the A-C view, highlighting SOX9 and PDGFRα-positive chondrocytes within the longitudinal lingual hyaline cartilage. Furthermore, Panels **G**-**I** depicted the circular lingual hyaline cartilage (CHC), chondrocytes (Ch), and perichondrium (F). Like the longitudinal cartilage, chondrocytes within the circular hyaline cartilage also express SOX9 and PDGFRα. White dotted lines have been added to panels A, D, and G to delineate the shape of the longitudinal and circular hyaline cartilage. Scale bars: A-C = 100 μm, D-F = 50 μm, G-I = 100 μm

Our study revealed a significant presence of SOX9 and PDGFRα-positive chondrocytes in the longitudinal cartilage in the tongue body (Fig. 9A). Upon closer examination at higher magnification, these chondrocytes exhibited strong SOX9 and PDGFRα expression (Fig. 9D). Furthermore, we observed the expression of SOX9 and PDGFRα in the perichondrium surrounding all cartilage types (Fig. 9). Remarkably, the circular hyaline cartilage located in the root of the tongue also displayed intense SOX9 and PDGFRα-positive chondrocytes, along with perichondrial expression (Fig. 9G). Of particular note, the co-expression of SOX9 and PDGFRα, indicated by the yellow color (Fig. 9C, F, I), underscores potential interactions between these markers within the chondrocytes of both longitudinal and circular cartilage.

### CD20 immunofluorescence reveals B Cell lymphocyte distribution in the lingual tonsil of the red-eared slider

**Fig. 10:**
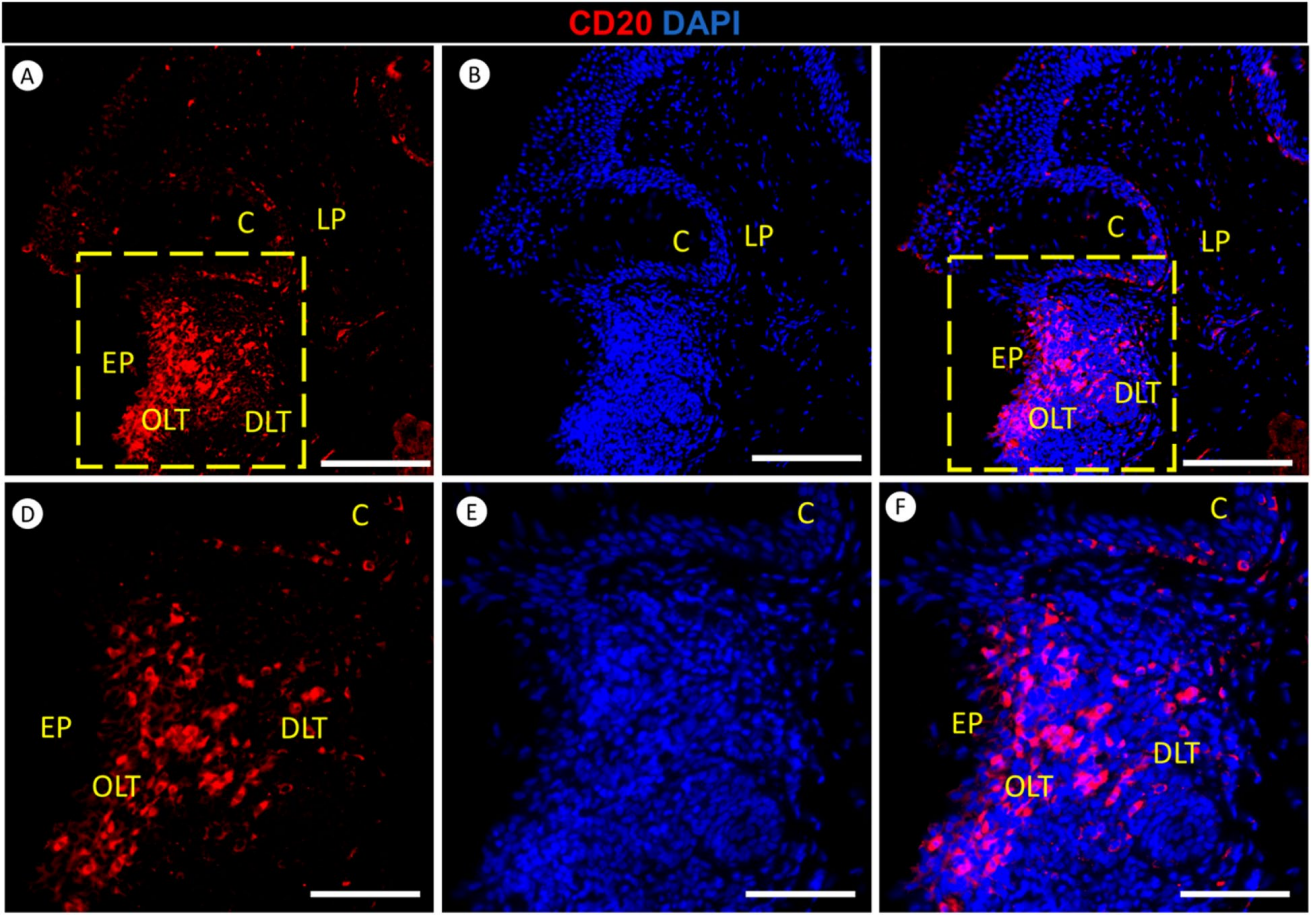
Immunofluorescence staining of CD20 (Red) and DAPI (Blue) in the lingual tonsil of the red-eared slider. Panels **A**-**C** provide a lower magnification view, while Panels D-F present a higher magnification view, revealing the distribution of lymphoid tissue in the tongue of the red-eared slider. Lymphocytes (L) are found in various locations, including diffuse lymphoid tissue (DLT), dorsal organized lymphoid tissue (OLT) in lingual epithelium (EP), crypt (C), and sparsely within the lamina propria of dense irregular connective tissue (LP). Scale bars: A-C = 100 μm, D-F = 50 μm

CD20 immunofluorescence staining was employed in our study to investigate the distribution of B cell lymphocytes within the lymphoid tissue of the red-eared slider’s tongue root. B-cells are a type of lymphocyte, a white blood cell involved in the immune system, specifically responsible for producing antibodies. CD20 immunofluorescence revealed the distribution of B-cell lymphocytes within various lymphoid tissue compartments in the red-eared slider’s lingual tonsil (Fig. 10). Higher concentrations of lymphocytes were observed in the diffuse lymphoid tissue and the dorsal organized lymphoid tissue regions in lingual epithelium. Conversely, fewer lymphocytes were noted in the crypt and lamina propria of dense irregular connective tissue compartments within the tongue’s root tissue Fig. 10).

### A versatile role of α-SMA in the lingual tissue of the red-eared slider

**Fig. 11:**
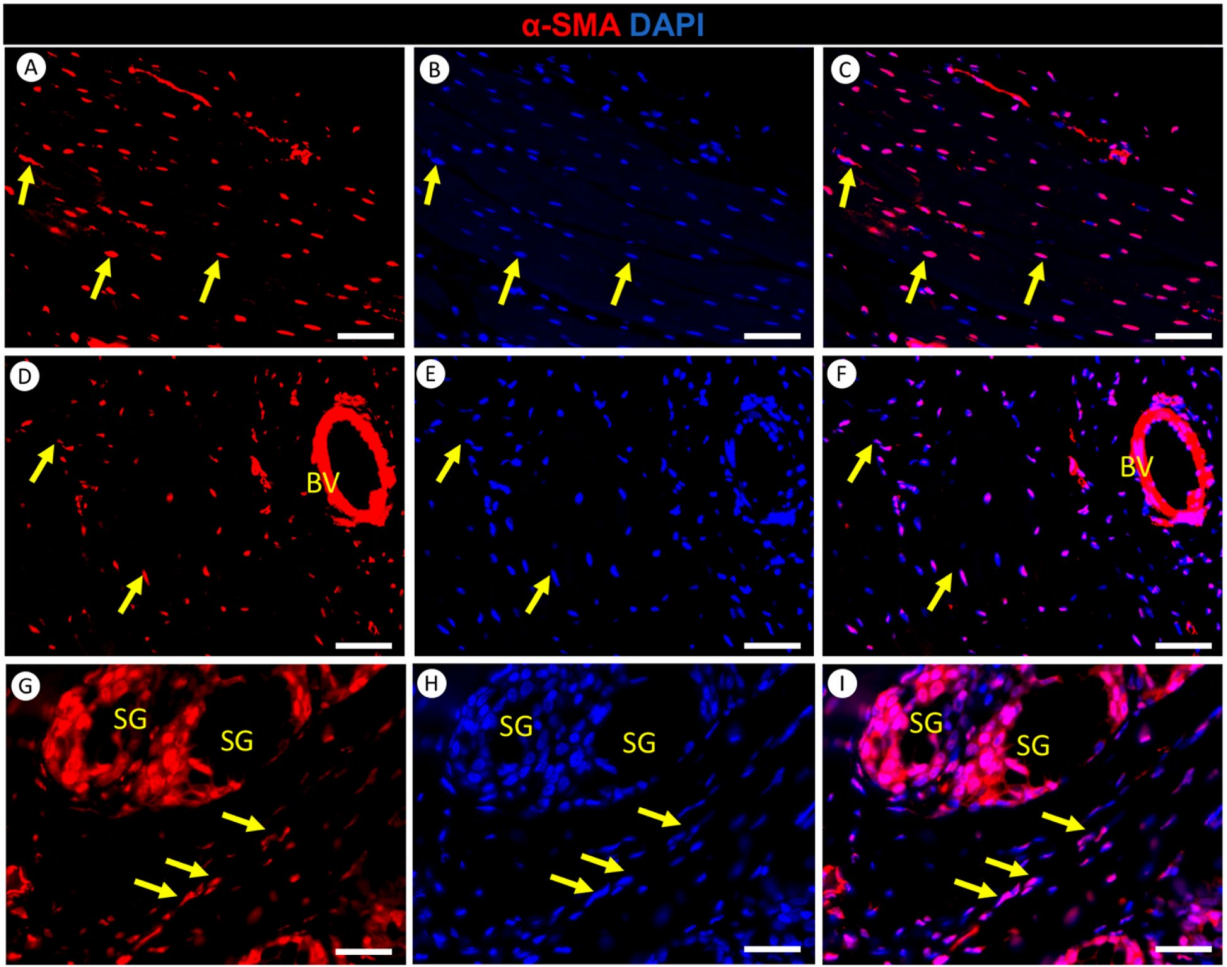
Immunofluorescence staining of α-SMA (Red) and DAPI (Blue) in the tongue of the red-eared slider. Panels **A**-**C** illustrate α-SMA expression in myofibroblasts (yellow arrows) within the intrinsic muscle of the tongue. Panels **D**-**F** depict α-SMA expression in the smooth muscle of blood vessels (BV) and myofibroblasts (yellow arrows). Panels G-I depict α-SMA expression in the smooth muscle of the salivary glands (SG) and myofibroblasts (yellow arrows). Scale bars: A-F = 50 μm, G-I = 25

α-SMA is a marker primarily used to identify smooth muscle cells. However, it’s essential to note that α-SMA can also be present in various cell types beyond smooth muscle, including myofibroblast cells. In our examination of red-eared slider tongue tissue, we observed the presence of α-SMA in myofibroblast cells (Fig. 11). These myofibroblast cells exhibited a spindle-shaped morphology, a characteristic feature of smooth muscle cells. Myofibroblast cells play specialized roles in tissue repair and remodeling (Fig. 11). Additionally, we detected α-SMA in the smooth muscles of both lingual blood vessels (Fig. 11D-I) and the lingual salivary gland (Fig. 11G-I). α-SMA forms a distinct concentric layer encircling the vessel walls and salivary gland within the lingual tissue (Fig. 11D-I).

## Discussion

Turtle species have successfully adapted to various ecological conditions, spanning from aquatic to fully terrestrial environments. These adaptations are closely linked to their phylogenetic history and are evident in multiple anatomical features, especially within the oropharynx. The oropharynx plays a crucial role in various essential functions such as respiration, olfaction, thermoregulation, defense mechanisms, courtship displays, and feeding [47].

The tongue is a key player in the feeding process of turtles and reflects their ecological success. This is particularly evident in the red-eared slider, a semiaquatic omnivorous species, which aligns with the ecological adaptation theory proposed by [48]. The tongue is divided into three distinct parts: the apex, body, and root, a characteristic shared with other turtle species such as basal tortoises [49], Egyptian tortoises [50], and other semiaquatic turtles [22]. In contrast to some earlier findings, the red-eared slider’s tongue is muscular, firmly anchored in the submandibular space, and posteriorly protected by the glottic bulge. This differs from the description of fish tongues, which lack voluntary muscles but have a mucosal elevation [51]. Similarities with other vertebrates are noted in the red-eared slider’s tongue structure [48].

The shape and coloration of the red-eared slider’s tongue exhibited variation, resembling a pale arrowhead. This observation correlates with the species’ preference for aquatic habitats and animal-based diets instead of plant-based foods, often resulting in non-movable, pale-colored tongues [9]. This tongue immobility contrasts turtles like common musk turtles, which can protrude their tongues outside the oral cavity [47]. The shape of the lingual apex, such as the pointed, non-bifurcated apex in the red-eared slider, plays a crucial role in food uptake, especially in land tortoises with protrudable tongues. This morphology enhances food capture and processing by increasing the tongue’s surface area [52].

Lingual papillae cover the dorsal surface of the tongue and significantly contribute to feeding strategies. In the red-eared slider, these papillae differ from purely aquatic turtles by being ridge-like, stacked closely with minimal interpapillary fissures, and acting as a cohesive unit. This adaptation may explain the smaller lingual mass in semiaquatic red-eared sliders. Aquatic turtles with reduced tongue mobility compensate for this limitation with well-developed hyoid and adductor muscles [21]. The shape and arrangement of lingual papillae reflect the mutual relationship between tongue function and the environment in which turtles live and feed. The red-eared slider’s larger lingual mass and immovable tongue suggest a preference for aquatic feeding, with little interest in land-based food sources [20]. Exceptions to this pattern, such as freshwater omnivorous terrapins and giant Asian pond turtles, are noted for their ability to protrude their tongues and feed on land [20, 47].

The lingual apex displays ridge-like papillae that fuse into a single unit with surface taste pores, while minimal interpapillary space is observed except at the lingual apex’s end. Towards the lingual body, papillae have a blunt and broad dorsal surface, with taste pores located at the lateral sides. This contrasts with observations in semiaquatic omnivorous *Heosemys grandis*, which have clear interpapillary spaces and no taste pores in their lingual apex [20]. The posterior portion of the tongue, near the laryngeal mound, exhibits papillae with rough surfaces featuring numerous microvilli but no observed taste pores. Similar roughness due to microvilli is found in other reptiles, such as basal tortoises [52]. In contrast, land tortoises have rough lingual papillae due to numerous keratinocytes [49]. The red-eared slider’s lingual papillae, characterized by ridge-like bluntness, resemble those of other generalized omnivorous turtles (*Malayemys subtrijuga, Pelusios castaneus, and Rhinoclemmys pulcherrima*) [52–54].

Tongue mobility plays a critical role in feeding mechanisms. The red-eared slider’s immovable tongue relies solely on suction mechanisms for bolus transportation, a trait supported by previous research [21, 55]. The fixed position of the tongue’s two posterior wings, anchored by the lingual frenulum and the ligament of the tongue’s root, contributes to its immobility, consistent with observations in other aquatic feeding turtles [21]. The sublingual space, housing sublingual glands, is relatively small in the red-eared slider due to its predominantly aquatic lifestyle, reducing the need for excessive moisturizing [56]. In purely aquatic turtles, lingual papillae are wholly lost [53].

Lingual papillae play a pivotal role in feeding strategies, with a combination of mechanical and gustatory (sensory) papillae found in both examined species. Fungiform papillae, especially at the lingual body, contain taste buds responsible for gustatory perception. They increase the tongue’s surface area, enhancing food friction and contributing to the sensory process of taste selection [20]. Moreover, mucous cells within the fungiform papillae facilitate taste sensation by adhering to taste buds [57]. The lamina propria in the tongue houses Ruffini nerve corpuscles, serving sensory, mechanical, and secretory functions. This aligns with previous research [58]. Both species exhibit well-developed tongue musculature, which acts as the tongue’s core and contributes to its mobility and the compression of lingual glands [59].

This study introduces the presence of mucosal-associated lymphoid tissue in the lamina propria of the red-eared slider’s tongue, characterized by an organized aggregation of lymphoid tissue close to the mucosal surface. This is like observations in the esophagus and intestine of turtles [60]. This lymphoid tissue likely plays an immunological role in protecting the mucosal surface and may serve as lingual tonsils or their equivalent. Lingual cartilage extends along the entire length of the red-eared slider’s tongue, which is unusual for reptiles. In contrast, avian tongues often contain hyaline cartilage [61].

We investigated the distribution of Vimentin in the red-eared slider turtle’s tongue, emphasizing its role in the structure and function of this sensory organ. Vimentin-positive cells were identified in crucial areas, including the lamina propria and stroma, underscoring their importance in maintaining the tongue’s architecture for sensory functions. Furthermore, the detection of vimentin in taste buds within fungiform papillae, suggesting a potential role in taste perception. This aligns with prior research by [26] and [62], who also found Vimentin expression in taste buds, highlighting its significance in taste sensation across different species.

The presence of synaptophysin within nerve bundles surrounding papillae and within taste buds in the red-eared slider turtle’s tongue underscores the outcomes of our investigation. This highlights the essential role of synaptophysin in regulating neural signaling and synaptic communication and shaping the sensory processes in the turtle’s tongue. The presence of synaptophysin in nerve bundles emphasizes the importance of neural transmission in taste perception, and its localization within taste buds reveals the complexity of synaptic interactions underlying taste sensation. These findings provide valuable insights into this unique species’ intricate taste perception process. Our results are consistent with previous research by [27], who also observed synaptophysin in taste buds in rats, specifically in vallate papillae and nasoincisor ducts. Furthermore, our findings align with the work of [28], who identified synaptophysin expression in taste receptor cells of Wistar rats. Their research suggests that synaptophysin may be involved in transmitting taste signals to the brain, as it is located in bitter, umami, sweet taste receptor cells, as well as presynaptic cells forming synapses with taste nerves. This suggests a crucial role for synaptophysin in the transmission of all five basic tastes. Our results indicate that synaptic interactions among the cells within taste buds are integral to the intricate process of taste perception in red-eared slider turtles.

The identification of CD34 and PDGFRα in the lamina propria and stroma of papillae within the red-eared slider’s tongue suggests the activation of signaling pathways associated with tissue maintenance and repair. This underscores the critical roles of CD34 and PDGFRα in sustaining the papillae’s structure and function. These findings contribute to overall mucosal tissue health in the turtle’s tongue, underscoring the dynamic nature of the lamina propria. Various tissue-regulating factors collaborate to maintain papillae functionality, ensuring proper tongue function in sensory perception and food processing.

Our findings are consistent with the research conducted by [46], where CD34 and PDGFRα were employed as markers for identifying telocytes. Their observations revealed the co-expression of CD34 and PDGFRα, confirming the presence of telocytes within the stromal framework [30]. described how CD34+/PDGFRα + telocytes formed unique spatial networks in the tongue’s lamina propria, located beneath the lingual epithelium. Notably, these telocytes did not exhibit immunopositivity for α-SMA. The co-expression of CD34 and PDGFRα in our results strongly indicates the presence of telocytes in the tongue of the red-eared slider. This discovery suggests a potential association of telocytes with crucial roles in tongue maintenance and repair processes. Anticipating the roles often attributed to CD34 and PDGFRα, we envisage that these telocytes may play a key role in orchestrating tissue integrity, supporting repair mechanisms, and contributing to the overall structural and functional resilience of the tongue. To explore these potential roles further, a detailed analysis using transmission electron microscopy is planned for future investigations.

SOX9 and PDGFRα-positive chondrocytes were unveiled within the hyaline cartilage of the red-eared slider turtle’s tongue, emphasizing the crucial role of this tissue in supporting various functions related to feeding, communication, and potentially other ecological adaptations. The intensity of marker expression suggests that these chondrocytes are actively involved in maintaining the tongue’s cartilage’s structural and functional aspects, emphasizing the unique and specialized nature of this adaptation in red-eared slider turtles. The expression of SOX9, a critical transcription factor in chondrogenesis, within the cartilage of the turtle’s tongue provides valuable insights into the regulatory mechanisms governing cartilage formation in non-mammalian species. While SOX9’s role in cartilage formation in various species is well-documented [31], its presence within specific cartilaginous tissues, such as the lingual cartilage in turtles, prompts a comparative analysis with tissues associated with bone formation in other organisms.

No prior studies describe the different marker expressions within hyaline cartilage in the tongue of turtles, and we are the first to undertake this exploration. In mammals, SOX9 plays a pivotal role in the early stages of chondrogenesis, contributing to the formation of cartilage templates that eventually develop into the skeleton [31]. However, the expression and regulation of SOX9 in bone-forming tissues are more complex. SOX9 has been identified in osteochondral progenitors [63], and it is essential for endochondral ossification during long bone development [64]. Its presence in these contexts highlights its dual role in both cartilage and early bone formation.

The detection of PDGFRα in hyaline cartilage of red-eared slider turtle’s tongue is consistent with [33], who found that PDGFRα plays a significant role in chondrocranial cartilage development. They investigated the development of craniofacial cartilage in mammals, a critical aspect of skull formation. Their study revealed that PDGFRα influences chondrocyte progenitor formation in embryonic mesenchymal stem cells and promotes their proliferation. However, in our study, we detected PDGFRα in the hyaline cartilage of adult red-eared sliders, emphasizing its role in maintaining cartilage health in mature turtles. Our results provide valuable insights into the presence of SOX9 and PDGFRα in chondrocytes, suggesting their role in the structural and functional aspects of the lingual hyaline cartilage. The co-expression of SOX9 and PDGFRα may signify their collaborative involvement in maintaining the integrity and homeostasis of cartilage tissue.

CD20-positive B-cell lymphocytes were found in the tongue root of red-eared slider turtles. This discovery holds significant anatomical and functional implications, as lymphocytes play a crucial role in the immune system. Their presence in this area suggests potential immune responses or surveillance activity. Given that the tongue root is a critical site where foreign particles, pathogens, or antigens may contact the turtle’s mucosal tissue during feeding or ecological interactions, the presence of B-cell lymphocytes, characterized by CD20 expression, serves as a defense mechanism, safeguarding the turtle against potential threats and contributing to its overall immune function. Our research addresses a notable research gap, as no prior studies have explored CD20 expression in any type of turtle. Therefore, our investigation sheds light on the distribution of B-cell lymphocytes in these unique reptiles. Our findings align with the work of [65], who conducted a study to assess CD20 expression in healthy canine spleens, normal canine peripheral blood cells, and canine lymphoma cells. They found that CD20 is expressed on both normal and malignant canine lymphocytes. Their study aimed to evaluate the potential utility of CD20 as a diagnostic tool for detecting normal lymphocytes and as a therapeutic target. Our findings highlight variations in lymphocyte distribution, indicating diverse concentrations of immune cells within the microenvironment of the tongue.

α-SMA was revealed within myofibroblasts in the red-eared slider’s tongue, and this distribution extended to the smooth muscle surrounding lingual blood vessels and the salivary gland. These findings highlight the diverse roles of α-SMA, including maintaining tissue integrity, facilitating muscle function, and potentially regulating vascular tone within the turtle’s tongue microenvironment. In contrast to our findings, CD34/α-SMA double immunostaining used but did not identify a myofibroblast-like telocyte subtype within the human tongue stroma [30]. However, it is worth noting that reported positive α-SMA signals in human lingual vascular smooth muscle and myoepithelial cells within the secretory salivary gland units, which aligns with our findings [30].

## Conclusion

The tongue of a red-eared slider has a fixed position that has no ability to protrude outside the mouth or meet the external environment. Our study explained that the fixation of the tongue with its pointed shape in a red-eared slider is due to the lingual frenulum and the ligament of the root of the tongue that fix the two wings caudally; thus, the tongue isn’t a food prehension tool in this turtle, but it depends on suction feeding through creating a negative pressure inside the oropharyngeal cavity. The dorsal surface of the tongue is rough and fully occupied by papillae which are ridge-like, and the interpapillary spaces are less clear. The taste pores and taste buds are noticed on the surface of the fungiform papillae. The ventral surface of the tongue is coated with stratified squamous epithelium with lots of mucous cells. Our study employed immunofluorescence techniques to investigate the cellular and molecular aspects of the red-eared slider’s tongue, known for its remarkable adaptations. This research illuminated the complex anatomy and physiology of this species, addressing a crucial knowledge gap. Vimentin highlighted taste bud cells, synaptophysin unveiled taste bud and nerve bundle characteristics, CD34 and PDGFRα labeled stromal cells within lingual papillae, and SOX9 and PDGFRα identified chondrocytes in the tongue’s cartilage. CD20 marked B-cell lymphocytes in the lingual tonsil, while α-SMA delineated myofibroblasts and smooth muscle surrounding blood vessels and salivary glands. Our study contributes to turtle biology, enhancing our understanding of the species’ remarkable adaptations in its ecological niches.

## Data Availability

The datasets used and/or analyzed during the current study are available from the corresponding author on reasonable request.
